# Computing With Networks of Chemical Oscillators and its Application for Schizophrenia Diagnosis

**DOI:** 10.3389/fchem.2022.848685

**Published:** 2022-02-16

**Authors:** Ashmita Bose, Jerzy Gorecki

**Affiliations:** Institute of Physical Chemistry, Polish Academy of Sciences, Warsaw, Poland

**Keywords:** chemical computing, oscillations, Oregonator model, networks, genetic optimization, schizophrenia, EEG signal

## Abstract

Chemical reactions are responsible for information processing in living organisms, yet biomimetic computers are still at the early stage of development. The bottom-up design strategy commonly used to construct semiconductor information processing devices is not efficient for chemical computers because the lifetime of chemical logic gates is usually limited to hours. It has been demonstrated that chemical media can efficiently perform a specific function like labyrinth search or image processing if the medium operates in parallel. However, the number of parallel algorithms for chemical computers is very limited. Here we discuss top-down design of such algorithms for a network of chemical oscillators that are coupled by the exchange of reaction activators. The output information is extracted from the number of excitations observed on a selected oscillator. In our model of a computing network, we assume that there is an external factor that can suppress oscillations. This factor can be applied to control the nodes and introduce input information for processing by a network. We consider the relationship between the number of oscillation nodes and the network accuracy. Our analysis is based on computer simulations for a network of oscillators described by the Oregonator model of a chemical oscillator. As the example problem that can be solved with an oscillator network, we consider schizophrenia diagnosis on the basis of EEG signals recorded using electrodes located at the patient’s scalp. We demonstrated that a network formed of interacting chemical oscillators can process recorded signals and help to diagnose a patient. The parameters of considered networks were optimized using an evolutionary algorithm to achieve the best results on a small training dataset of EEG signals recorded from 45 ill and 39 healthy patients. For the optimized networks, we obtained over 82% accuracy of schizophrenia detection on the training dataset. The diagnostic accuracy can be increased to almost 87% if the majority rule is applied to answers of three networks with different number of nodes.

## 1 Introduction

It is known that chemical reactions are responsible for acquiring information, transmitting it, and decision-making in living organisms. However, the number of man-written algorithms that can be efficiently executed using a chemical medium Adleman, (1994); Kuhnert (1986), Kuhnert et al. (1989); Steinbock et al. (1995); Agladze et al. (1997); Vazquez-Otero et al. (2012) is quite limited. We believe that the difficulties in practical applications of chemical computers are mainly caused by the fact that an efficient strategy for signal coding using a chemical medium has not been developed yet. The most popular approach to chemical computing imitates information processing with semiconductor devices. In semiconductor devices, information is binary coded in different values of electric potentials.

The success of semiconductor technology came as the consequence of the highly efficient realization of semiconductor binary logic gates. Such gates are characterized by a long time of error-free operation. The semiconductor gates can be assembled together inside an integrated circuit, producing more complicated information processing devices. The bottom-up design strategy Feynman et al. (2000) perfectly matches the technology; complex devices are made as a concatenation of simpler ones. On the contrary, chemical logic gates Toth and Showalter (1995); Steinbock et al. (1996); Sielewiesiuk and Gorecki (2001); Adamatzky et al. (2002) are not small nor fast. The time of their reliable operation is measured in hours, not in years. It seems very hard to make a chemical medium where millions of gates are combined together and work as planned for a long time.

Here we describe and discuss an example of chemical computation based on non-binary information coding. The presented results are based on numerical simulations of the time evolution of the considered computing medium. Such an approach has been motivated by similarities between nerve signals and propagating pulses in a spatially distributed medium in which Belousov-Zhabotinsky (BZ) reaction proceeds Gorecki (2015). The BZ-reaction is catalytic oxidation of an organic substrate in an acidic environment Belousov (1959); Zhabotinsky (1964); Field and Burger (1985); Epstein and Pojman (1994). Two stages of BZ reaction can be visually identified. One of these stages is the fast oxidation of the catalyst. The other is a slow reduction by an organic substrate. The color of the solution of BZ-medium reflects concentrations of catalyst in the oxidized and reduced forms. Therefore, the nonlinear behavior of the medium as oscillations between reduced and oxidized states, propagation of the region characterized by a high concentration of oxidized catalyst, or appearance of spatio-temporal patterns can be easily observed. In a spatially distributed medium, where BZ-reaction proceeds, a local excitation corresponding to the high concentration of *HBrO*_2_ can propagate in space in the form of the concentration pulse. This type of behavior resembles the propagation of nerve impulses in living organisms. As a result, the BZ-reaction has attracted attention as a medium for experiments with neuron-like chemical computing Adamatzky et al. (2005); Gorecka and Gorecki (2006). Within the most popular approach to chemical computing with BZ-medium, it is assumed that information is transmitted by propagating pulses of the oxidized form of catalyst. For a binary coding, the presence of a pulse represents the logic TRUE state, and the state with a low concentration of the catalyst in the oxidized form is the logic FALSE state Field and Burger (1985); Epstein and Pojman (1994).

If the ruthenium complex $\left(Ru{\left(bpy\right)}_{3}^{2+}\right)$ is used as the reaction catalyst, then BZ-reaction becomes photosensitive Kadar et al. (1997). Oscillations can be inhibited by light. For the same initial concentrations of reagents, the medium can oscillate at dark and show an excitable behavior at a low light intensity. And it converges to a steady-state when it is strongly illuminated.

In a medium with photosensitive BZ-reaction excitable channels in which signals can propagate can be formed by specific illumination of a spatially distributed medium. Using a suitable geometry of excitable and non-excitable channels, one can force an appropriate type of interactions between excitations and, for example, make a signal diode Agladze et al. (1996), a memory cell, or logic gates Adamatzky et al. (2005); Yoshikawa et al. (2009). However, in typical applications, such gates are big (with an area of about 1 cm^2^), and a single operation takes more than 10 s Epstein and Pojman (1994). Therefore, the bottom-up approach from gates to complex information processing tasks does not look promising if the binary is used with BZ-medium.

Recent studies have demonstrated that an oscillating BZ-reaction can be efficiently applied for information processing Smelov and Vanag (2018); Vanag VK. (2019); Proskurkin et al. (2020); Egbert, (2019); Duenas-Diez and Perez-Mercader (2019); Duenas-Diez and Perez-Mercader (2020). For example, it has been shown that a network of interacting chemical oscillators can be trained to perform classification tasks with a reasonable accuracy Gruenert et al. (2015); Gizynski and Gorecki (2017a). To illustrate the problem, let us consider a database *D*_*A*_ composed of *N* records:

$$D_{A}=\left\{\left(p_{n}^{1},p_{n}^{2},\ldots ,p_{n}^{k},q_{n}\right),n=1,N\right\}\;.$$ (1)

The records have a form of (*k* + 1) tuples $\left(p_{n}^{1},p_{n}^{2},\ldots ,p_{n}^{k},q_{n}\right)$ , where the first *k* elements are predictors and the last element (*q*_*n*_) is the discrete record type. A classifier of *D*_*A*_ is supposed to return the correct data type if the predictor values are used as the input.

We can easily define the predictor values and the corresponding record type for many life-inspired classification problems, but usually we do not know how to relate both quantities. For example, the needs of medical diagnostic belong to such class of problems. The input information (the predictor values $\left(p_{n}^{1},p_{n}^{2},\ldots ,p_{n}^{k}\right)$ ) are collected from several medical tests on the patient ^#^n. On this ground, we are expected to conclude if the patient is healthy or not (the value of *q*_*n*_). Our knowledge of the relationship between input (results of medical tests) and output (patient’s health condition) is based on previously accumulated examples. For such problems, the top-down design strategy Gizynski et al. (2016) of a chemical computing medium seems to be more beneficial than the bottom-up one.

Let us assume we have selected a classification problem to be solved. In the following text, it is the determination if a patient has schizophrenia or not. To apply the top-down strategy, we should decide about the medium that is supposed to perform the classification. Here we assume that a network of interacting chemical oscillators can approximately solve the determination of schizophrenia problem. We do not know which network is the best for this task, so we considered a few simple networks illustrated in Figure 2. We selected these particular networks because our previous studies indicated that for a fixed number of modes, a large number of connections in increases the accuracy. All networks can include nodes (oscillators) of two types Gizynski and Gorecki (2017a); Gizynski et al. (2016); Gizynski and Gorecki (2016). The input nodes belong to the first type. They are used to introduce the values of predictors into the network. If a node is assigned as the input of the *i*th predictor, then its oscillations are suppressed for the time interval of length related to the value of *p*_*i*_. There are also so-called normal oscillators that are inhibited for a fixed time that is not related to the predictor value. These normal oscillators moderate interactions between oscillators in the medium and optimize the network to solve a specific problem. The time intervals during which their activity is suppressed do not depend on the input. These time intervals define the program executed by the network. For the analysis presented below, we assume that the output information is coded in the number of oscillation cycles observed on a given node. The choice of the output oscillator follows directly from the network optimization. The complete definition of a computing network includes the number of oscillators in the network, their types, locations, the information about the time intervals they are active, and the information about interactions between oscillators.

Obviously, a network with randomly selected parameters has a small chance to work as a good classifier. We have to optimize its parameters (i.e., to teach a network) to perform the selected function. Teaching means that we need a teacher, and in our optimization, it is a specific database *T*_*A*_ that contains diagnostic results and information if a patient is ill or healthy EEG (n. d.). In the following, we do not change the number of oscillators in a network nor modify the geometry of interactions between them.

The application of the top-down strategy to the considered networks means that the parameters such as locations of the input and normal oscillators, inhibition times for the normal oscillators, the method for inputting the values of predictors, or the parameters of reactions responsible for interactions between oscillators are the subjects of optimization. The optimization is supposed to achieve the best match with a representative (training) dataset of cases *T*_*A*_. We have found Gruenert et al. (2015); Gizynski and Gorecki (2017a); Gizynski et al. (2016); Gizynski and Gorecki (2016) that evolutionary optimization oriented on obtaining the best classifier for a representative training dataset of the problem can lead to a computing network that performs the anticipated task with reasonable accuracy.

In previous papers on chemical database classifiers Gruenert et al. (2015); Gizynski and Gorecki (2017a); Gizynski et al. (2016); Gizynski and Gorecki (2016) an oversimplified event-based-model reflecting the basic features of the oscillator time evolution and of interactions between oscillators coupled by mutual activations was used. The event-based-model divides an oscillation cycle into three phases: excitation, refractory and responsive phase. It also assumes a sharp difference between these phases. An oscillator in the refractory phase is not susceptible to stimulations by interacting oscillations. However, the event-based-model allows for the excitation of an oscillator in the responsive phase that is in contact with an excited oscillator. In this paper, we consider a more realistic model. We represent the time evolution of an individual oscillator using the two-variable Oregonator model I. R. Epstein and Pojman (1994); Field and Noyes (1974) of the photosensitive Belousov-Zhabotinsky (BZ) reaction.

If we neglect interactions with the other oscillators of the network, then equations describing the time evolution of *j*th oscillator are:

$$\begin{array}{ccc} \frac{du_{j}}{dt} & = & \frac{1}{\varepsilon }\left(u_{j}-u_{j}^{2}-\left(fv_{j}+\phi_{j}\left(t\right)\right)\frac{u_{j}-q}{u_{j}+q}\right) \end{array}$$ (2)

$$\begin{array}{ccc} \frac{dv_{j}}{dt} & = & u_{j}-v_{j} \end{array}$$ (3)

Where the variables *u*_*j*_ and *v*_*j*_ represent concentrations of an activator (*U*_*j*_) and an inhibitor (*V*_*j*_) for proceeding reactions. The parameter *ɛ* sets up the ratio of time scales for variables *u* and *v*, *q* is a scaling constant, and *f* is the stoichiometric coefficient. We used the same values of model parameters for all oscillators in the network in our simulations: *ɛ* = 0.2, *q* = 0.0002, and *f* = 1.1. The parameters of the Oregonator model were fixed and did not undergo optimization.

If we assume that the time evolution of oscillators is described by a model of photosensitive BZ-reaction, then oscillators can be individually controlled by illumination, and we include this feature into the considered model as the time-dependent function *ϕ*_*j*_(*t*) in Eq. 2. The time-dependent function *ϕ*_*j*_(*t*) that describes the influence of illumination on an oscillator is proportional to the light intensity. We considered *ϕ*_*j*_(*t*) in the form:

$$\phi_{j}\left(t\right)=0.1\cdot \left(1.001+\tanh\left(-10\cdot \left(t-t_{illum}\left(j\right)\right)\right)\right.$$ (4)

**FIGURE 1:**
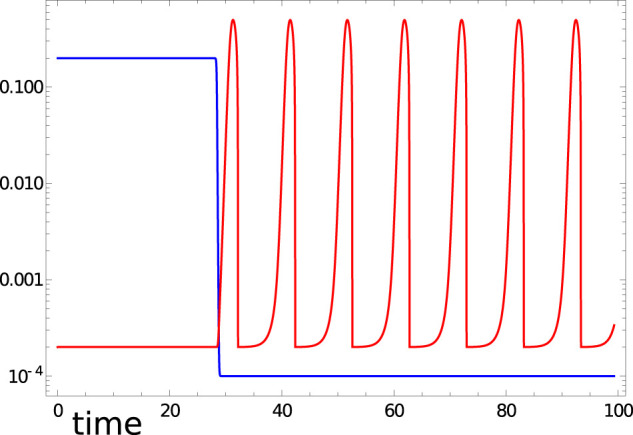
Illumination *ϕ*_*j*_(*t*) (blue curve) and time evolution of *u*_1_(*t*) (red curve) in the time interval [0,99.3] predicted by Eqs 2, 3 with the considered Oregonator parameters and *t*_*illum*_ (1)=28.4.

In this definition *t*_*illum*_(*j*) > 0 defines illumination of the *j*th oscillator. In the time interval [0, *t*_*illum*_(*j*) − *δ*] (*δ* = 0.1) the value of *ϕ*_*j*_(*t*) is high (∼0.2) . The Oregonator model with parameters given above predicts a stable steady state corresponding to *u*_*j*_ = 0.0002 and *v*_*j*_ = 0.0002. For long times (*t* > *t*_*illum*_(*j*) + *δ*) the value of *ϕ*_*j*_(*t*) approaches 0.0001 what corresponds to oscillations with the period of approximately 10.8 time units. The value of *δ* describing the speed of transition between the steady state and oscillations can be reduced by increasing the multiplier under tanh () function. In order to extract the answer of a classifier we consider the number of activator maxima within the time interval *Z* = [0, *t*_max_]. It can be noticed that Eq. (4) has a physical meaning for any value of *t*_*illum*_(*j*). If *t*_*illum*_(*i*) < 0 then *ϕ*_*i*_(*t*) is small and the oscillator ^*#*^ *i* is active during the whole observation interval. When *t*_*illum*_(*k*) > *t*_max_ than *ϕ*_*k*_(*t*) is large and the oscillator ^*#*^ *k* is blocked within *Z* and does not oscillate. Figure 1 shows the time evolution of activator *u*_1_(*t*) predicted by Eqs 2 and 3 with *t*_max_ = 99.3 and *t*_*illum*_ (1) = 28.4 (cf. Table 3). As seen for such parameters, oscillations restart just after *t*_*illum*_ (1), and the system produces seven activator maxima within the observation time interval.

In the investigated networks the values of *t*_*illum*_(*j*) for normal oscillators were fixed. On the other hand, if an oscillator was considered as the input of *p* ^*i*^ (*i* = 1, 2) then *t*_*illum*_(*j*) was an affine function of *p* ^*i*^ (cf. Eq. 7). Such function is defined by two parameters *t*_*start*_ and *t*_*end*_ and it has the form:

$$t_{illum}\left(j\right)=t_{start}+\left(t_{end}-t_{start}\right)\cdot p^{i}$$ (5)

We assumed that *t*_*start*_ and *t*_*end*_ are the same for all predictors in the considered schizophrenia records (*p* ^1^ and *p* ^2^).

**FIGURE 2:**
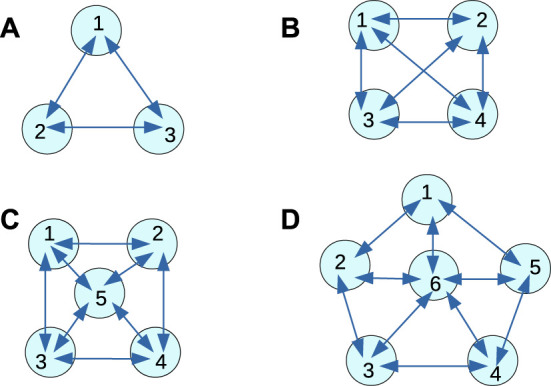
Geometries of oscillator networks considered for the schizophrenia diagnosis. The disks represent individual oscillators that can act as input oscillators or normal ones. Arrows show interactions among the oscillators. The numbers are used to mark individual oscillators in the following description of results. **(A)** 3-oscillator network, **(B)** 4-oscillator network, **(C)** 5-oscillator network, **(D)** 6-oscillator network.

**FIGURE 3:**
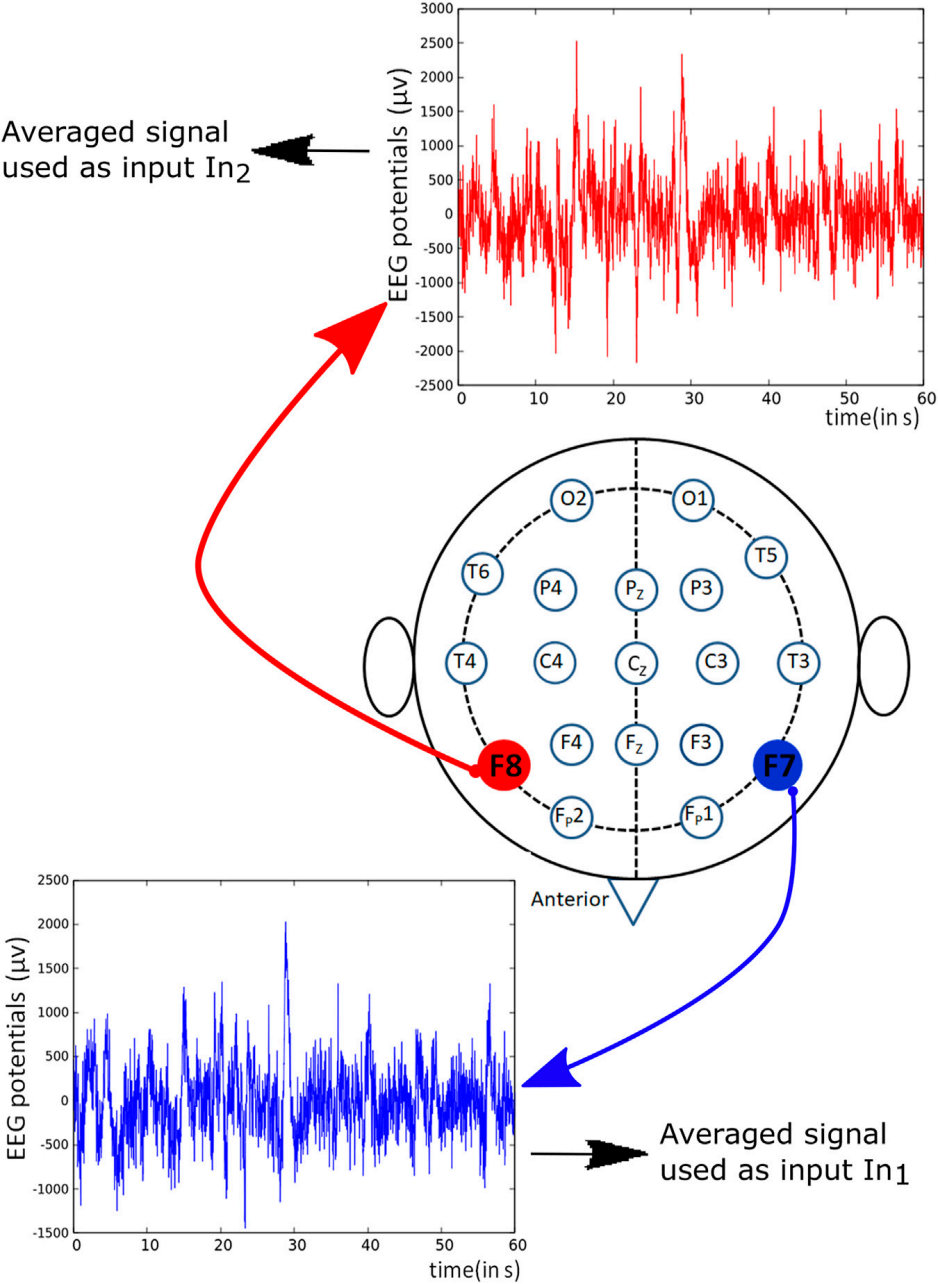
Schematic representation of positions of different electrodes used for recording EEG potentials. The potentials derived from the marked red channels were used to generate predictors in the training dataset *T*_*S*_.

It was demonstrated that even a small network composed of 16 or fewer oscillators, with the time evolution of mutual excitations described by the event-based-model could be used to diagnose if a cancer cell is malignant or benign Gizynski and Gorecki (2017a). In this report, we concentrate on designing a network of oscillators that can determine whether the patient has schizophrenia. Schizophrenia is one of the most common forms of psychotic behavior. The patients experience hallucinations, delusion, or disorganized speech. However, schizophrenia is difficult to detect Siebenhuhner et al. (2013). It is believed that the analysis of EEG signals recording brain activity can help to verify if a patient is ill or healthy Ben et al. (2007). The relevant EEG signals were recorded from electrodes placed in different parts of the scalp (see Figure 3). We postulate that a network of interacting chemical oscillators in the form presented in Figure 2 can process the information extracted from the EEG signals and help diagnose schizophrenia. Preliminary results obtained using the network illustrated in Figure 2D were reported in the extended abstract of the ASPAI 2020 Conference Bose and Gorecki (2020). For the database *T*_*A*_, available on the web EEG (n. d.) containing signals recorded on *N* = 84 patients, out of which *N*_*h*_ = 39 were healthy and the other had symptoms of schizophrenia (*N*_*s*_ = 45) the optimized classifier returned 82% correct answers. The extended study on the schizophrenia classifier in the network’s pentagon geometry (Figure 2D) was published in the International Journal of Unconventional Computing (IJUC) Bose and Gorecki (2021). In this IJUC paper, we also studied if the classification accuracy can be improved by dividing the whole recorded signal into 3 shorter (20 s long) parts and processing these shorter signals separately. Separated networks with Figure 2D geometry were optimized for each time subinterval within 500 generations of evolution. Next, the majority procedure was applied to obtain the final classification results. Such a method increased the classification accuracy of records in *T*_*A*_ to 90%. In the current report, we investigate if the classification accuracy can be improved by the network geometry. The results of schizophrenia diagnosis using classifiers with geometries illustrated in Figures 2A–C are new and have not been previously reported. Moreover, the presented results for the pentagon geometry differ from those published because the genetic optimization was performed for 260 more steps than in Bose and Gorecki (2021) and a new maximum of fitness was achieved during these additional steps. Therefore the pentagon-shaped classifier has a different structure than previously reported.

The manuscript is organized as follows. *Transformation of EEG Signals Into the Input Data* describes how the input data are extracted from the EEG signals. In *Numerical Model of Information Processing Network*, we present a numerical model for the simulation of network time evolution. *Network Optimization and Results* gives details of network optimization. The conclusions and suggestions for the future development of the networks for schizophrenia diagnosis are presented in the following *Conclusion and Discussion*.

## 2 Transformation of EEG Signals Into the Input Data

The considered networks were small, and there was no room for too many input variables because each predictor requires its input oscillator. We used the signals recorded from F7 and F8 channels marked red in Figure 3 as the inputs for schizophrenia detecting networks described below. Such choice is motivated by previous studies indicating that the signals obtained from the frontal lobe of the brain reveal the difference in the brain activity between a schizophrenic patient and a healthy subject Ben et al. (2007).

The EEG signals were recorded with a sampling rate of 128 Hz for 1 min. The medical EEG test of each patient produced 16 data files corresponding to signals recorded on different electrodes. Each data file contained *K* = 7,680 values of recorded potential (in *μV*). The time between consecutive potential values is Δ*t* = 7.812 5 *ms*. Let *V* ^*l*^ (*n*, *k*) denote the potential recorded for *n*th patient, on the *l*th electrode and at the time *t*_*k*_ = *k* ⋅Δ*t*. To reduce the size of input data, we averaged the recorded signals. Therefore, each signal was trimmed to a single number. We assumed that time average signals provide us with a sufficient amount of information to diagnose schizophrenia. The averaged potentials were defined as:

$$x_{n}^{l}=\overset{K}{\underset{k=0}{\sum }}V^{l}\left(n,k\right)$$ (6)

Next, the time averaged potentials recorded for the whole set of patients were normalized. We introduced:

$$\mu^{l}=\frac{1}{N}\overset{N}{\underset{n=1}{\sum }}x_{n}^{l}$$

and

$$\sigma^{l}=\sqrt{\frac{1}{N-1}\overset{N}{\underset{i=1}{\sum }}{\left(x_{n}^{l}-\mu^{l}\right)}^{2}}$$

The values of predictors $p_{n}^{1}$ and $p_{n}^{2}$ for the patient *n* were defined as:

$$p_{n}^{1}=\frac{x_{n}^{F7}-\mu^{F7}}{\sigma^{F7}},p_{n}^{2}=\frac{x_{n}^{F8}-\mu^{F8}}{\sigma^{F8}}\;\;$$ (7)

In the above equations *μ* ^*F*7^ = 7.724 *μV*, *μ* ^*F*8^ = 2.46 *μV*, *σ* ^*F*7^ = 20.3 *μV* and *σ* ^*F*8^ = 15.10 *μV*.

**FIGURE 4:**
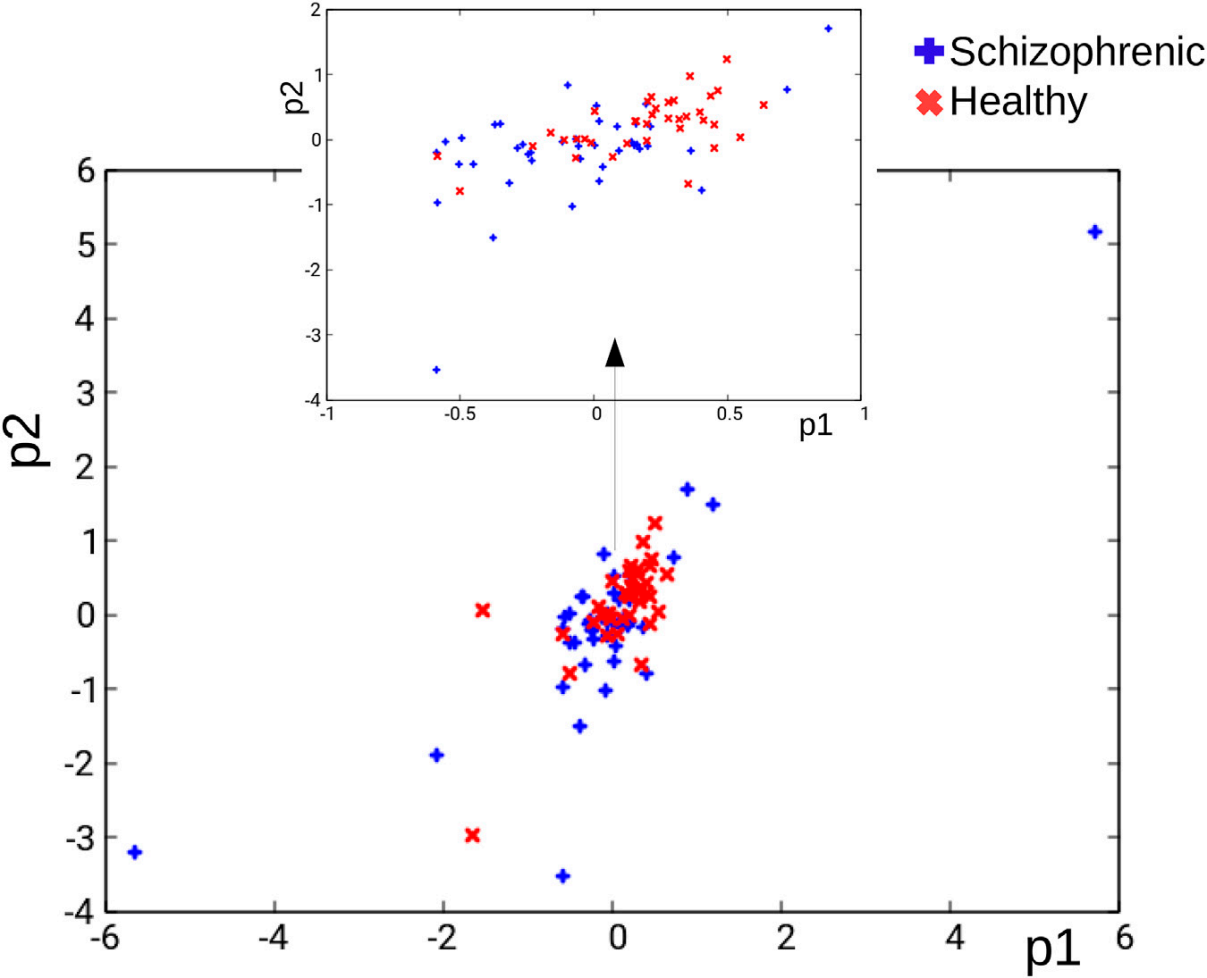
The distribution of records in the *T*_*S*_ database in the (*p*_1_, *p*_2_) coordinates. Blue and red crosses correspond to schizophrenic and healthy cases, respectively.

As a result, the problem of schizophrenia diagnosis is reduced to the best classification of the training dataset: $T_{S}=\left\{\left(p_{n}^{1},p_{n}^{2},q_{n}\right),n=1,N\right\}$ where the record type *q*_*n*_ = 0 for a schizophrenic patient and *q*_*n*_ = 1 for a healthy subject. The distribution of records in the *T*_*s*_ database in the (*p*_1_, *p*_2_) coordinates is illustrated in Figure 4. Blue and red crosses correspond to schizophrenic and healthy cases, respectively. It can be seen that the points corresponding to different cases are not separated, which makes their classification difficult.

In our study, we also considered predictors generated using combinations of signals recorded on other electrodes, but for those, the classification accuracies on corresponding datasets were lower.

## 3 Numerical Model of Information Processing Network

The time evolution of reactions proceeding in a single oscillator was described by the two-variable Oregonator model (Eqs 2 and 3). We assumed that interactions between the oscillators ^#^k and ^#^j appeared as the results of reactions involving the activators *U*_*k*_ and *U*_*j*_ of these oscillators:

$$\begin{array}{ccc} U_{j}+B_{j}\to U_{k}+C_{k} & & \end{array}$$ (8)

$$\begin{array}{ccc} U_{k}+B_{k}\to U_{j}+C_{j} & & \end{array}$$ (9)

with identical reaction rate constant *k*_*B*_.

We also assumed that the activator of each reaction could spontaneously decay in the binary reaction:

$$U_{j}+D_{j}\to products$$ (10)

with the rate constant *k*_*D*_. In the equations above symbols *B*, *C* and *D* denote other molecules involved in these reactions. Reactions (8–10) involving species *B*, *C* and *D* are formally introduced to explain chemical communication between nodes and to justify the mathematical description of it.

Therefore, the changes in concentrations of *U*_*k*_ and *U*_*j*_ as the result of reactions (8 and 9) are:

$$\begin{array}{ccc} \frac{du_{j}}{dt} & =-k_{B}b_{j}u_{j} & \end{array}$$ (11)

$$\begin{array}{ccc} \frac{du_{k}}{dt} & =-k_{B}b_{k}u_{k} & \end{array}$$ (12)

and the changes in concentration of *U*_*j*_ as the result of reaction (10) is:

$$\frac{du_{j}}{dt}=-k_{D}d_{j}u_{j}$$ (13)

In Eqs 11–13 *b*_*j*_, *b*_*k*_ and *d*_*j*_ denote concentrations of *B*_*j*_, *B*_*k*_ and *D*_*j*_, respectively. We assume that these concentrations were high with respect to concentrations of activators involved and the same for all oscillators. Therefore, the concentrations of *B*, *C*, and *D* were regarded as constant during the network evolution, and there is no need to include them in the model of network evolution. Let us introduce symbols *α* and *β* defined as: *α* = *k*_*D*_ *d*_*j*_ and *β* = *k*_*B*_ *b*_*j*_. Keeping in mind that values of *α* and *β* can be modified by concentrations of *B*_*j*_ and *D*_*j*_, we can treat them as free parameters that can be easily adjusted. Therefore, the values of *α* and *β* can be included in the optimization procedure. The same mathematical description of interactions between nodes applies for controlled exchange of reaction mixtures between nodes and outflow of activator in a system with immobilized catalyst.

Within our model the time evolution of the network is described by the following set of kinetic equations:

$$\begin{array}{ll} \frac{du_{j}}{dt} & =\frac{1}{\varepsilon }\left(u_{j}-u_{j}^{2}-\left(fv_{j}+\phi_{j}\left(t\right)\right)\frac{u_{j}-q}{u_{j}+q}\right)\\ & -\left(\alpha +\beta \underset{i=1,m}{\sum }s_{j,i}\right)u_{j}+\beta \left(\underset{i=1,m}{\sum }s_{j,i}u_{i}\right) \end{array}$$ (14)

$$\frac{dv_{j}}{dt}=u_{j}-v_{j}$$ (15)

The last two terms in Eq. 14 represent the coupling in between *i*th and *j*th oscillators and the activator decay. The symbols *s*_*j*,*i*_ are defined as:

*s*_*j*,*i*_ = 0 if *j* = *i* or if *j* ≠ *i* and oscillators ^*#*^ *j* and ^*#*^ *i* do not interact,

*s*_*j*,*i*_ = 1 if *j* ≠ *i* and oscillators ^*#*^ *j* and ^*#*^ *i* do interact.

The set of Eqs 14 and 15 describes the network evolution after all parameters characterizing the medium including *t*_*illum*_ for all oscillators are known.

A classifier is supposed to produce an answer within a finite time. However we do not know it. Therefore, the time *t*_max_ that defines the interval of time for which the network evolution is observed [*Z* = (0, *t*_max_)] is one of the optimized parameters of a classifier. We postulate that information about patient health is extracted from the number of activator maxima recorded on a selected oscillator of the network, during the time interval *Z*. In order to find which oscillator should be used as the output one we calculate the mutual information *I* (*G*; *O*_*j*_) Cover and Thomas (2006) between the discrete random variable *G* of record types in the training dataset *T*_*S*_ (*G* = {*q*_*n*_, *n* = 1, *N*}) and the discrete random variable *O*_*j*_ of the number of activator *u*_*j*_ maxima *o*_*j*_(*n*) observed on the *j*th oscillator in the network when the predictors of *n*th database record are used as the network input (*O*_*j*_ = {*o*_*j*_(*n*), *n* = 1, *N*}). The mutual information *I* (*G*; *O*_*j*_) can be calculated as:

$$I\left(G;O_{j}\right)=H\left(G\right)+H\left(O_{j}\right)-H\left(G,O_{j}\right)$$ (16)

where *H* () is the Shannon information entropy Shannon (1948) and the discrete random variable (*G*, *O*_*j*_) = {(*q*_*n*_, *o*_*j*_(*n*)), *n* = 1, *N*}. The oscillator ^*#*^ *i* for which the mutual information between *G* and *O*_*i*_ is maximal is used as the network output. The mutual information calculated for the output oscillator was considered as the measure of network fitness:

$$Fitness={max}_{j}I\left(G;O_{j}\right)$$ (17)

It can be expected that in the majority of cases the optimization based on the mutual information leads to a classifier with the highest accuracy Gorecki (2020).

## 4 Network Optimization and Results

### 4.1 Network Optimization

The network parameters as locations of input and normal oscillators, *t*_max_, *t*_*start*_, *t*_*end*_, the values of *t*_*illum*_(*j*) for normal oscillators and the rates *α*, *β* were subject of optimization. Following the idea of information coded in spikes Quiroga et al. (2009); Ghosh-Dastidar and Adeli (2009); Vidybida (2011) and the design of chemical classifiers described in Gruenert et al (2015) we optimized the system parameters using an evolutionary algorithm Koza (1989); Fogel (1994). In our calculations, the population of 200 networks was considered. In the beginning, the population of networks was randomly generated. The fitness of each network was calculated using the whole training dataset *T*_*S*_ as defined in Eq. 17.

The next generation of classifiers also consisted of 200 elements. It included 2% of the fittest networks from the previous generation that were copied without changes. The remaining 98% elements of the next generation were offsprings created by recombination and mutation operations applied to oscillators from the top 40% networks of the previous population. For recombination, two networks were selected and randomly separated into two parts. The separation into parts was identical for both networks. Next, an offspring was generated by combining one part of the first network with the other part of the second one. At this step, the function of an oscillator (input, normal) and illumination times of normal oscillators were copied to the offspring. The values of *t*_max_, *t*_*start*_, *t*_*end*_, *α* and *β* were randomly selected from the parent oscillators and copied to the offspring.

As the next step, mutation of the parameters of the newborn offspring was considered. We allowed for mutation on the rate of coupling between oscillators (*β*) and the rate of formation of product(*α*). The probability of mutation rate was 0.5 per step. The mutated values of *α* and *β* were the sum of a half of their old values and a random number.

We introduced no constraints on the oscillator types. The recombination procedure could produce an offspring without any input oscillators. It was also possible there were no normal oscillators in the offspring. The fate of such pathological offsprings was decided by its *Fitness*. If it was lower, then the offspring did not qualify into the 40% of networks, and the information about it was not used when the next generation of the networks was created.

The procedure described above was repeated for 1,000 generations. The classifiers discussed in the following were the fittest ones after completing the optimization.

### 4.2 Optimized Networks With Different Numbers of Chemical Oscillators for Schizophrenia Diagnosis

Now, let us present the optimized classifiers of geometries illustrated in Figure 2. We studied the time evolution of the networks by numerical solution of Eqs 14 and 15 using Cash-Karp R-K45 method William press et al (1992) with *h* = 10^–3^ time steps. The number of activator maxima *o*_*j*_(*n*) was calculated as the number of *u*_*j*_(*t*) maxima larger than 0.05, observed when the predictors $p_{n}^{1},p_{n}^{2}$ were used as the input.

**FIGURE 5:**
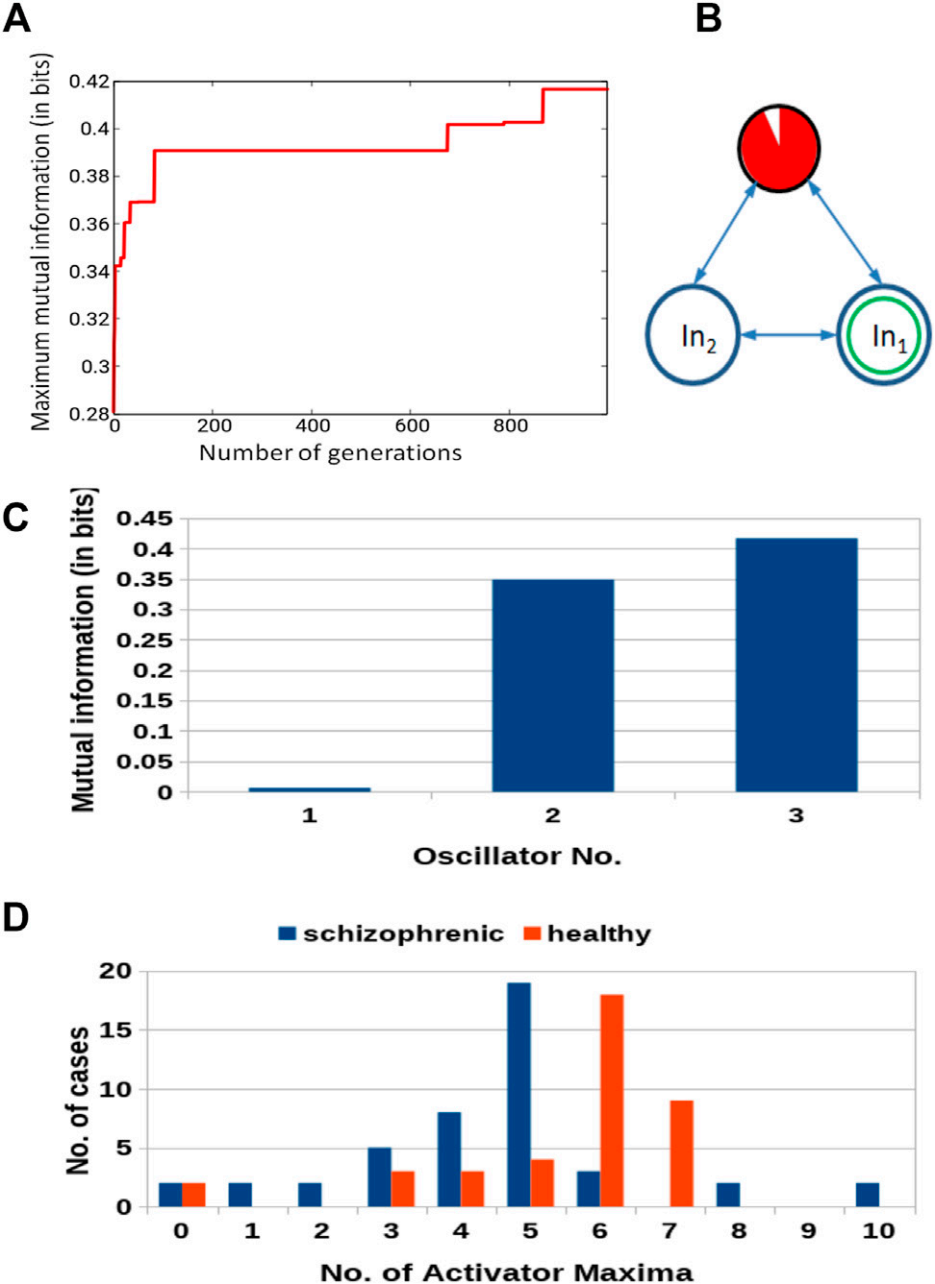
Results for 3-oscillator network (cf. Figure 2A): **(A)** The progress of optimization; the *Fitness* as a function of generation number. **(B)** The structure of the optimized network. The disk within a black circle is the normal oscillator. The ratio between the surface of the red shaded part and the disk surface represents the ratio between $t_{illum}^{j}$ and *t*_max_. *In*_1_ and *In*_2_ mark inputs for *p* ^1^ and *p* ^2^. The disk with the green circle inside is the output oscillator. **(C)** The mutual information *I* (*G*; *O*_*j*_) for *j* ∈{1,2,3}. The mutual information has the maximum at the oscillator ^#^3. **(D)** The distribution of the numbers of cases for which a given number of activator maxima was observed on oscillator ^#^3. Colors indicate records representing schizophrenic and healthy patients.

Figure 5A shows the progress of optimization as the function of number of generations for the network composed of three oscillators. The increase in *Fitness* is fast for the first few generations. Next, it changes into randomly distributed jumps with decreasing amplitude and frequency. Such dependence of the *Fitness* is typical for genetic optimization of classifiers Gruenert et al. (2015); Gizynski and Gorecki (2017a). The *Fitness* observed after 1,000 optimization steps was 0.417 bit. The parameters describing the best classifier are given in Table 1.

**TABLE 1:** Parameters of the optimized 3-oscillator network.

Parameter	Value
*t*_max_	99.8
*t*_*start*_	42.5
*t*_*end*_	6.3
*α*	0.89
*β*	0.36
*t*_*illum*_ (1)	92.5

**FIGURE 6:**
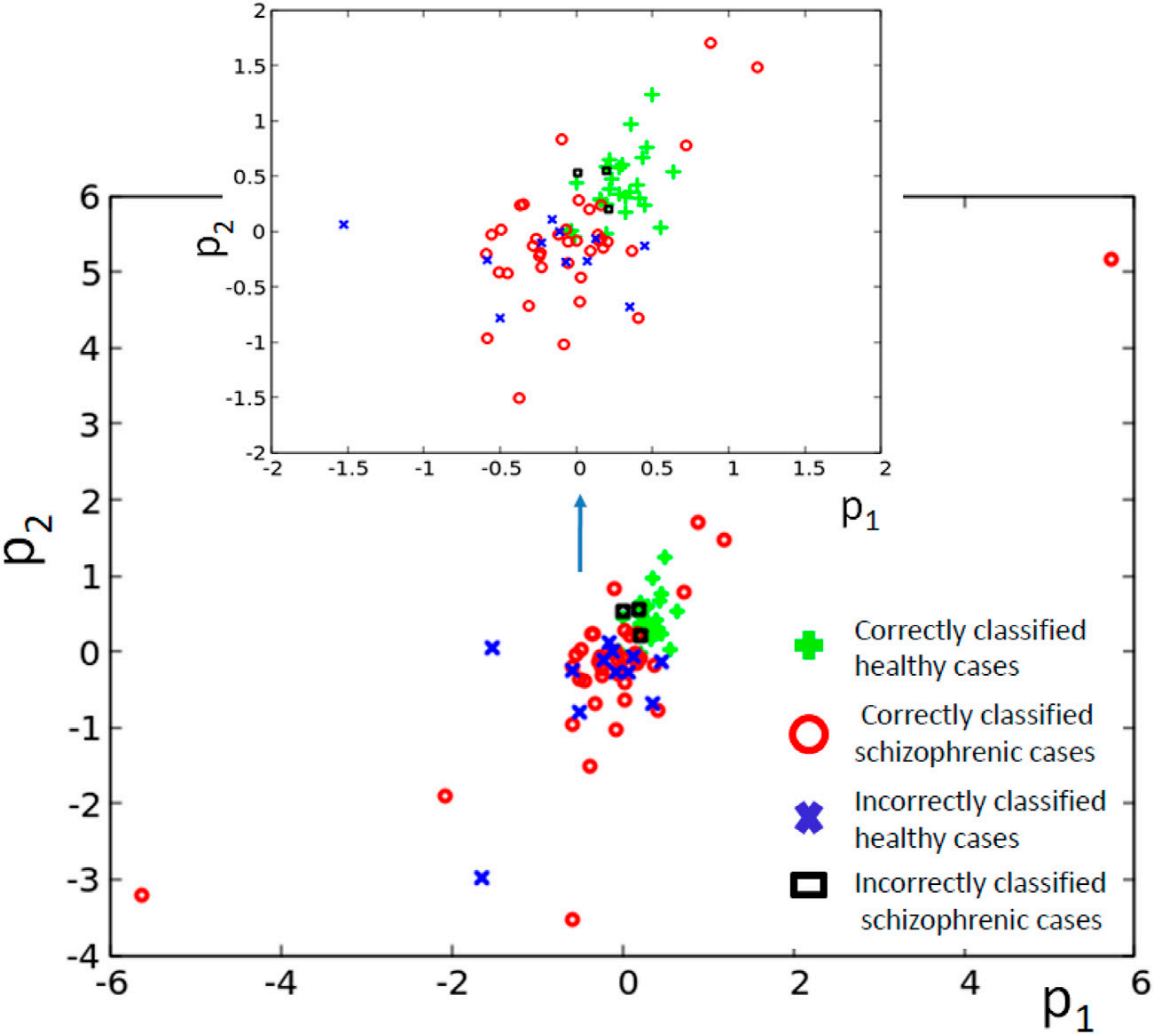
The distribution of correctly and incorrectly classified cases for 3-oscillator network (cf. Figure 2A) in the phase space (*p*_1_, *p*_2_).

Figure 5B illustrates the structure of the optimized classifier. It is interesting to notice that the normal oscillator remained non-active for the majority of the time when the network evolution was observed. This feature is reflected by the values of *I* (*G*; *O*_*j*_) shown in Figure 5C. The value of *I* (*G*; *O*_1_) is very small, which means that the activity of the oscillator ^#^1 gives little information about the patient health. The value of *I* (*G*; *O*_3_) = 0.417 is the maximum one; thus, the oscillator ^#^3 was selected as the output one. Figure 5D shows the distribution of numbers of activator maxima observed on the oscillator ^#^3 for schizophrenic and healthy patients. This result suggests the following classification rule: a patient is healthy if the number of activator maxima is six or seven. The observation of any other number of maxima diagnoses schizophrenia. The application of this rule gives 15 errors for 84 cases included in *T*_*S*_ (82% accuracy). Only three schizophrenic patients (of 45) are diagnosed as the healthy ones. It gives over 93% accuracy in detecting the illness. On the other hand, 12 healthy people (of 39) are diagnosed as schizophrenic ones (30% error). If these results are confirmed using a large dataset of cases, then the 3-oscillator classifier can detect healthy people with high accuracy, because the “healthyˮ answer of the classifier is incorrect in three of the total 30 answers (10%). On the other hand, if a person is diagnosed as “ill”, then such diagnosis can be wrong in 12 of 54 answers (over 22%). Therefore, the “illˮ diagnosis requires further investigation. Positions of correctly and incorrectly classified cases for 3-oscillator network (cf.1a) in the phase space (*p*_1_, *p*_2_) are shown in Figure 6.

**FIGURE 7:**
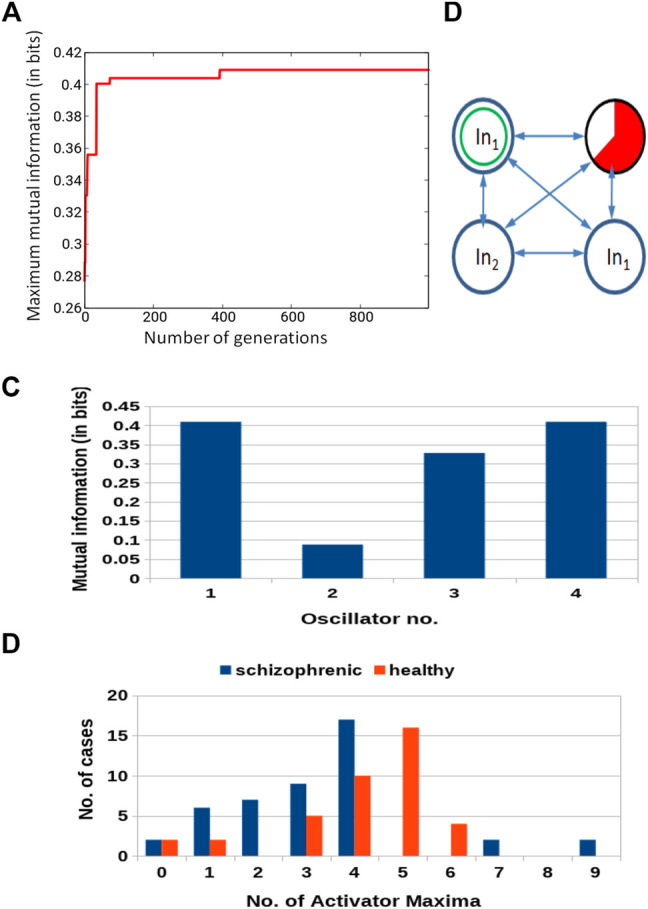
Results for 4-oscillator network (cf. Figure 2B): **(A)** The progress of optimization; the *Fitness* as a function of generation number. **(B)** The structure of the optimized network. Notation as in Figure 5B. **(C)** The mutual information *I* (*G*; *O*_*j*_) for *j* ∈{1,2,3,4}. The function *j* → *I* (*G*; *O*_*j*_) has the maximum at *j* =1. **(D)** The distribution of the numbers of cases for which a given number of activator maxima was observed on oscillator ^#^1 for records representing schizophrenic and healthy patients.

Similar results for optimization of the 4-oscillator classifier are illustrated in Figure 7A. The *Fitness* observed after 1,000 optimization steps was 0.409 bit. The parameters describing the best classifier are given in Table 2.

**TABLE 2:** Parameters of the optimized 4-oscillator network.

Parameter	Value
*t*_max_	94.1
*t*_*start*_	57.7
*t*_*end*_	11.5
*α*	0.61
*β*	0.36
*t*_*illum*_ (1)	59.6

**FIGURE 8:**
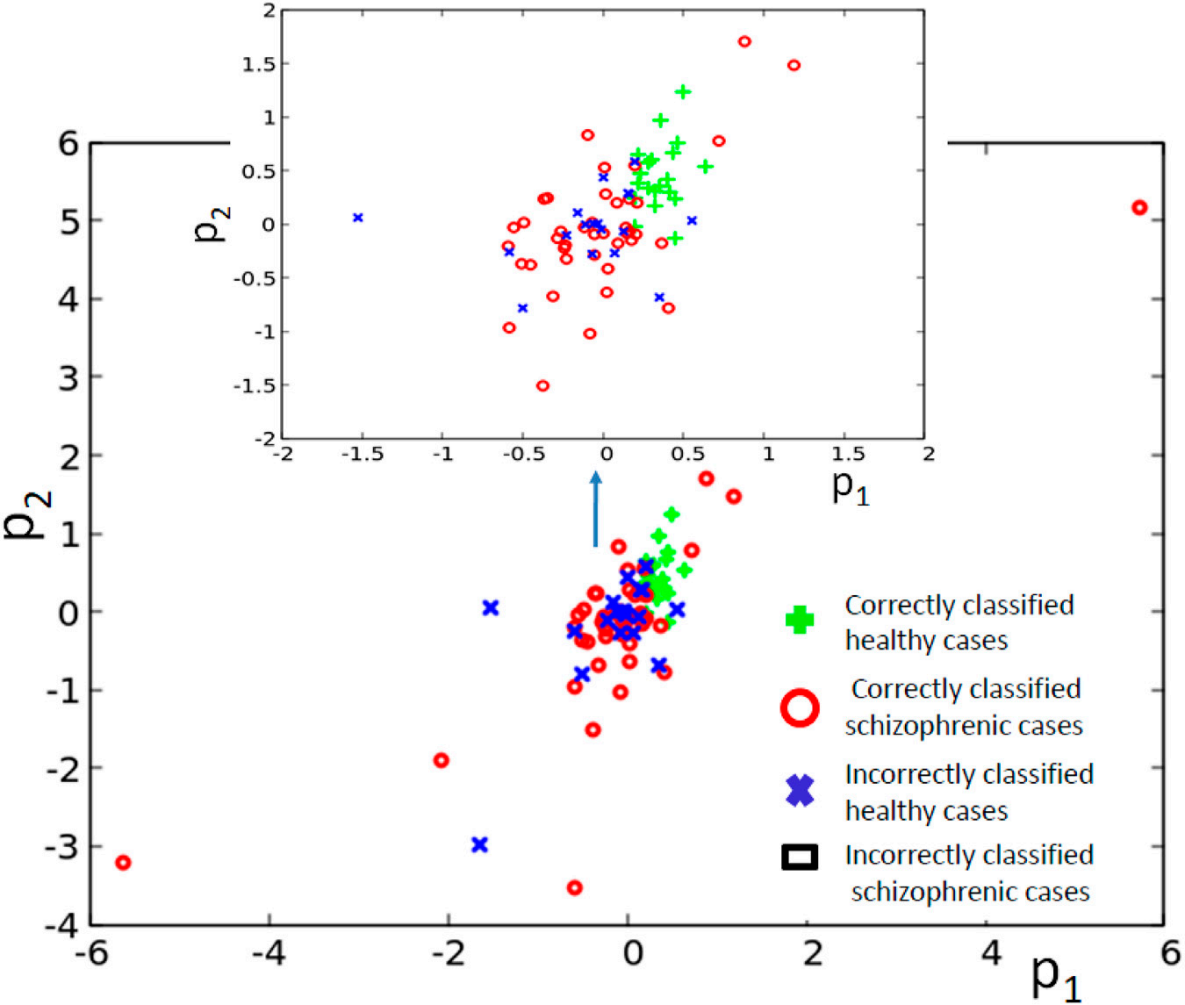
The distribution of correctly and incorrectly classified cases for 4-oscillator network (cf. Figure 2B) in the phase space (*p*_1_, *p*_2_).

Figure 7B illustrates the structure of the optimized classifier. There are two oscillators that act as inputs of the predictor *p* ^1^ and a single input for predictor *p* ^2^. Due to the network symmetry *I* (*G*; *O*_1_) = *I* (*G*; *O*_4_) (Figure 7C). These values (0.409 bit) are the maximum ones; thus, both oscillators ^#^1 and ^#^4 can be selected as the output one. In Figure 7B, we marked the first of them. Figure 7D shows the distribution of numbers of activator maxima observed on the oscillator ^#^1 for schizophrenic and healthy patients. The classification rule based on the majority of cases for a given number of activator maxima is: a patient is healthy if the number of activator maxima is five or six. The observation of any other number of maxima diagnoses schizophrenia. The application of this rule gives 19 errors for 84 cases included in *T*_*S*_ (77% accuracy). All incorrectly diagnosed patients are the healthy ones who are diagnosed as being schizophrenic. On the other hand, ALL schizophrenic patients were correctly diagnosed. Figure 8 presents locations of correctly and incorrectly classified cases for 4-oscillator network in the phase space (*p*_1_, *p*_2_).

**FIGURE 9:**
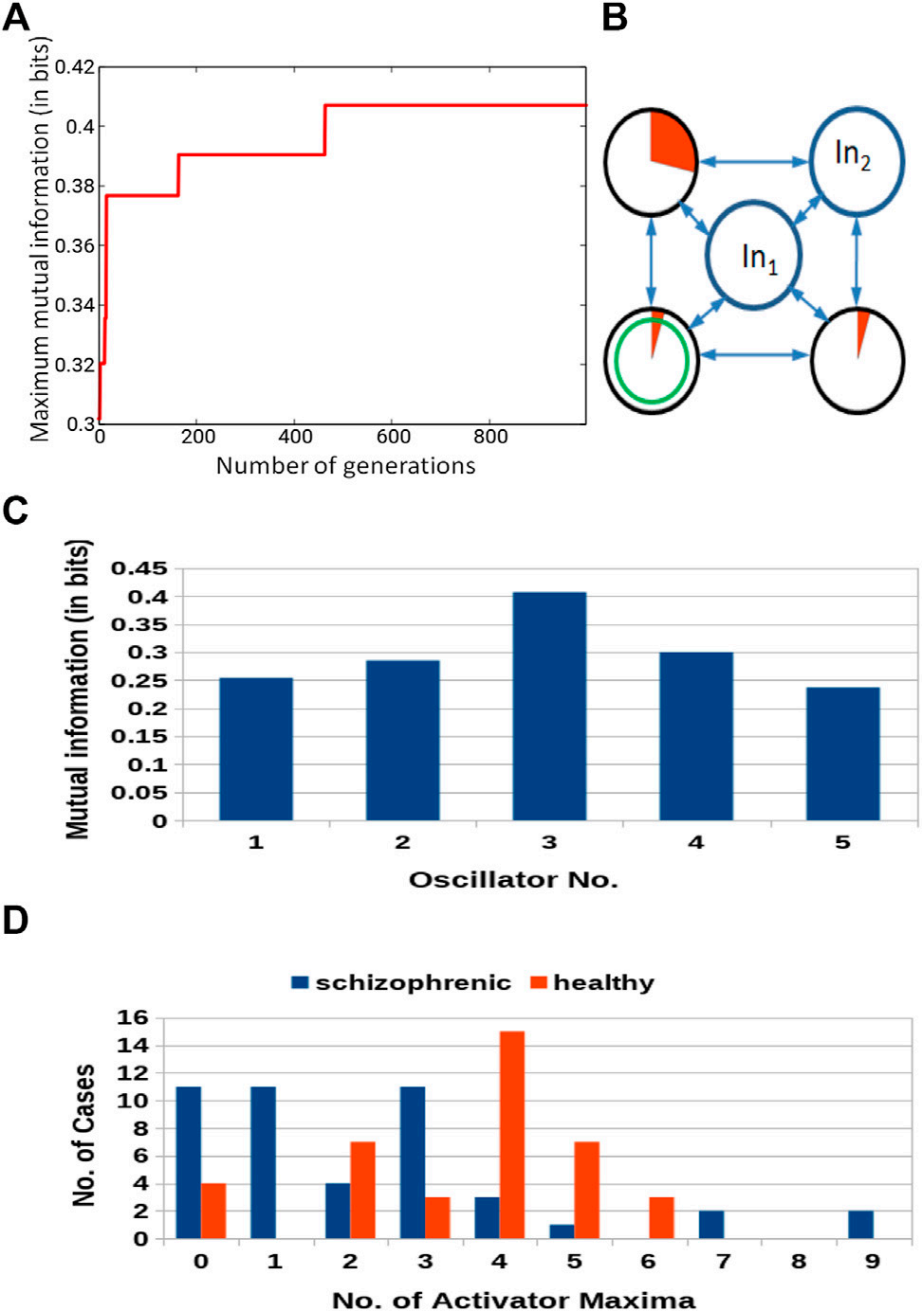
Results for 5-oscillator network (cf. Figure 2C): **(A)** The progress of optimization; the *Fitness* as a function of generation number. **(B)** The structure of the optimized network. Notation as in Figure 5B. **(C)** The mutual information *I* (*G*; *O*_*j*_) for *j* ∈{1,2,3,4,5}. The function *j* → *I* (*G*; *O*_*j*_) has the maximum at *j* =3. **(D)** The distribution of the numbers of cases for which a given number of activator maxima was observed on oscillator ^#^3 for records representing schizophrenic and healthy patients.

**FIGURE 10:**
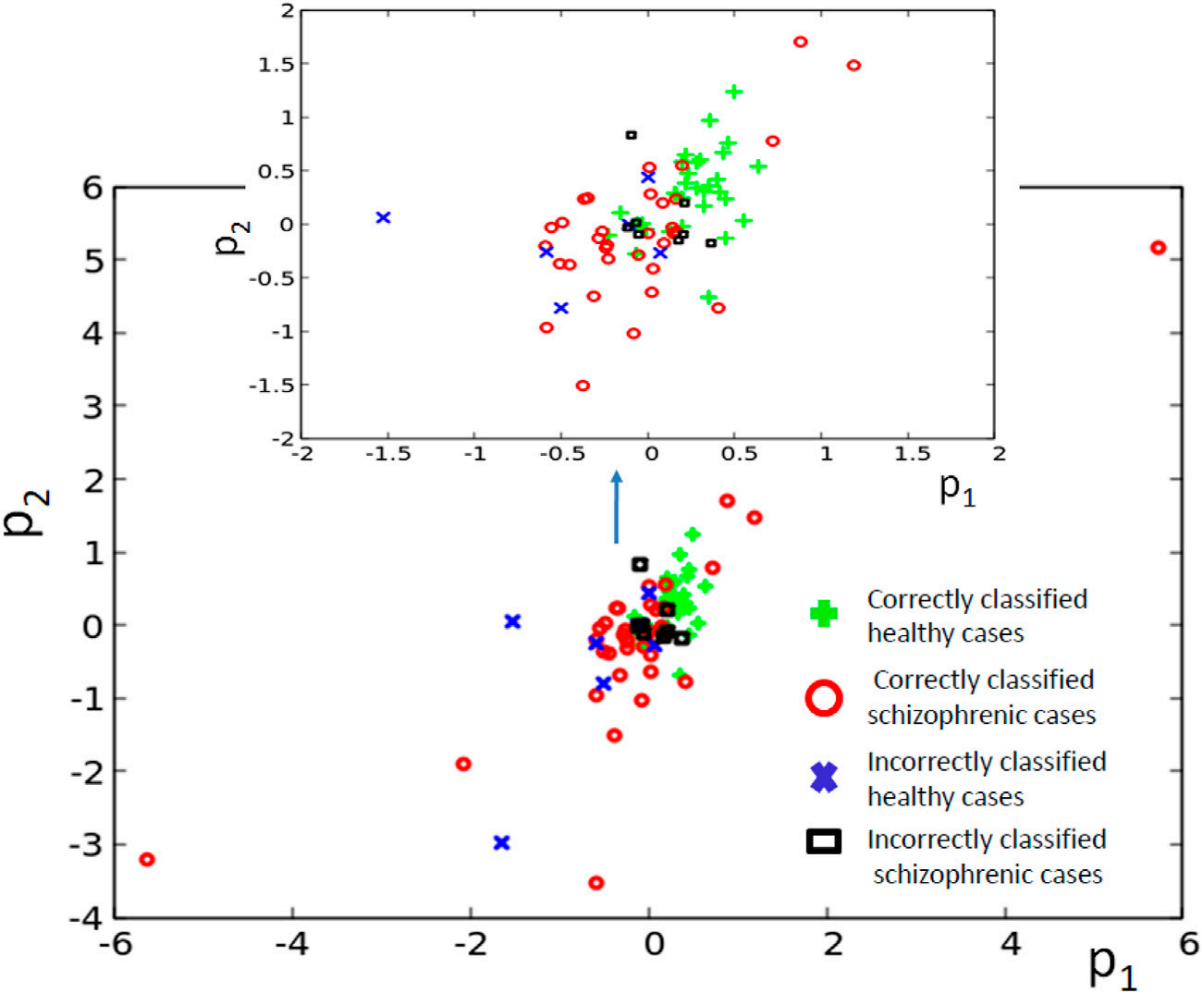
The distribution of correctly and incorrectly classified cases for 5-oscillator network (cf. Figure 2C) in the phase space (*p*_1_, *p*_2_).

The results for optimization of the 5-oscillator classifier are illustrated in Figure 9A. The *Fitness* observed after 1,000 optimization steps was 0.407 bit. The parameters describing the best classifier are given in Table 3. Figure 9B illustrates the structure of the optimized classifier. It is highly asymmetric and includes three normal oscillators. There are two oscillators that act as inputs of the predictor *p* ^1^ and a single input for predictor *p* ^2^. The highest value of *I* (*G*; *O*_*j*_) was observed for the oscillator ^#^3 (Figure 9C) that has no direct contact with the input of predictor *p* ^2^. Figure 9D shows the distribution of numbers of activator maxima observed on the oscillator ^#^3. As for the three- and four- oscillator cases the output oscillator does not generate small nor large numbers of activator maxima for healthy patients. The 5-oscillator network diagnoses a patient as a healthy one if the number of activator maxima is 2, 4, 5, or 6. The observation of any other number of maxima diagnoses schizophrenia. The application of this rule gives 15 errors for 84 cases included in *T*_*S*_ (82% accuracy). The schizophrenic patients are diagnosed with very similar accuracy as the healthy ones (82.2 vs. 82.1%). Correctly and incorrectly classified cases for 5-oscillator network are located in the phase space (*p*_1_, *p*_2_), as shown if Figure 10.

**TABLE 3:** Parameters of the optimized 5-oscillator network.

Parameter	Value
*t*_max_	99.3
*t*_*start*_	72.5
*t*_*end*_	13.8
*α*	1.11
*β*	0.23
*t*_*illum*_ (1)	28.4
*t*_*illum*_ (3)	4.64
*t*_*illum*_ (4)	4.64

**FIGURE 11:**
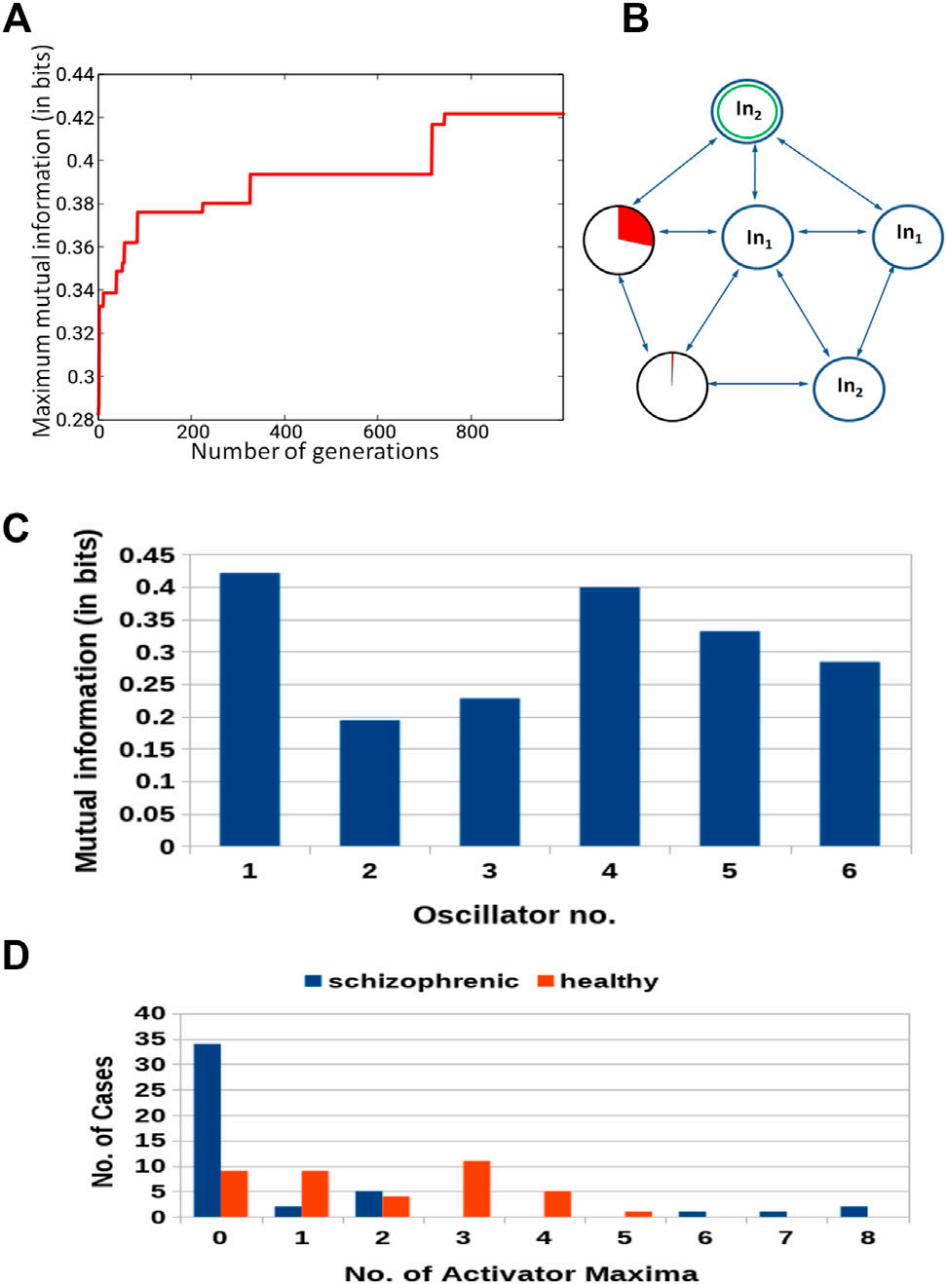
Results for 6-oscillator network (cf. Figure 2D): **(A)** The progress of optimization; the *Fitness* as a function of generation number. **(B)** The structure of the optimized network. Notation as in Figure 5B. **(C)** The mutual information *I* (*G*; *O*_*j*_) for *j* ∈{1,2,3,4,5,6}.The function *j* → *I* (*G*; *O*_*j*_) has the maximum at *j* =1. **(D)** The distribution of the numbers of cases for which a given number of activator maxima was observed on oscillator #1 for records representing schizophrenic and healthy patients.

**FIGURE 12:**
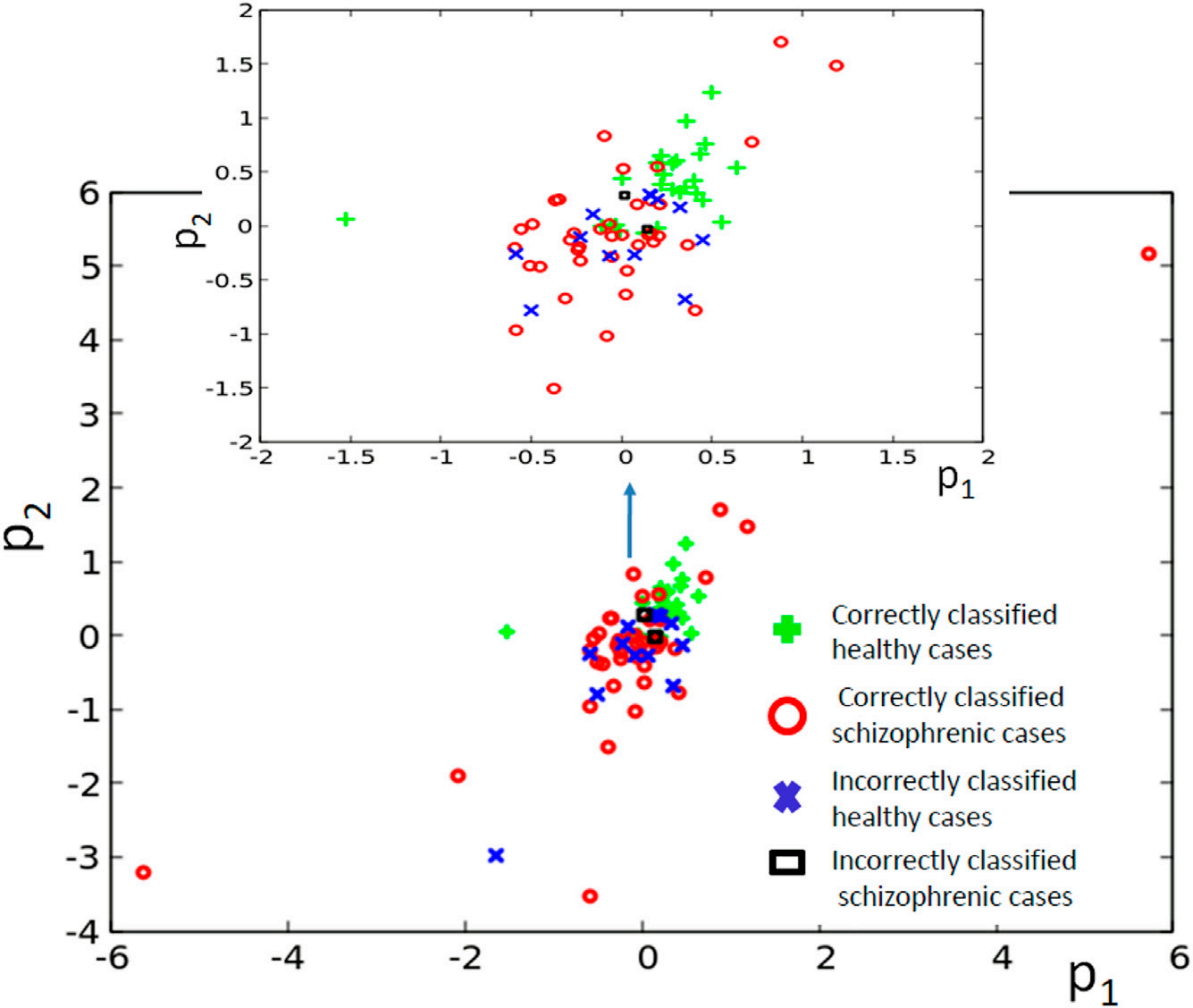
The distribution of correctly and incorrectly classified cases for 6-oscillator network (cf. Figure 2D) in the phase space (*p*_1_, *p*_2_).

The classifier made of six oscillators arranged in the geometry illustrated in Figure 2D was discussed in our abstract for the ASPAI 2020 Conference Bose and Gorecki (2020) and in Bose and Gorecki (2021). In both publications, the classifier was optimized for 740 generations, and the maximum *Fitness* was 0.416 bit. Figure 11 shows the results of optimization for 1,000 generations. The *Fitness* observed after 1,000 optimization steps was 0.422 bit. The parameters of the best classifier are given in Table 4. Figure 11B illustrates the structure of the optimized classifier. It is similar to that of the classifier reported in Bose and Gorecki (2020, 2021). In all optimized classifiers, there are two normal oscillators, two oscillators that act as inputs of the predictor *p* ^1^, and two oscillators representing inputs of *p* ^2^. In all classifiers, the input of *p* ^2^ was also the classifier output. Moreover, in the central oscillator was the input of *p* ^1^. However, there are also differences. In previously reported classifiers, inputs of *p* ^2^ were directly interconnected. In the structure shown in Figure 11B, they are separated by inputs of *p* ^1^. Figure 11D shows the distribution of numbers of activator maxima observed on the oscillator ^#^1. On its basis, we can deduce the following classification rule: a patient is healthy if the number of activator maxima is 1, 3, 4, or 5. The observation of any other number of activator maxima indicates that the patient is ill. Application of this rule gives 15 errors for 84 cases included in *T*_*S*_ (82% accuracy); thus, the accuracy is exactly the same as for the classifier reported in Bose and Gorecki (2021). For both classifiers, the structures of errors were similar. The previously reported classifier diagnosed incorrectly 12 of 39 healthy patients and three schizophrenic ones. The classifier reported in this paper diagnosed incorrectly 13 of 39 healthy patients and two who were ill. It is worth noticing that for the majority of schizophrenic cases, the optimized classifier in the geometry in Figure 11D did not produce a single activator maximum at the output oscillator. Figure 12 illustrates the positions of correctly and incorrectly classified cases for 6-oscillator network in the phase space (*p*_1_, *p*_2_).

**TABLE 4:** Parameters of the optimized 6-oscillator network.

Parameter	Value
*t*_max_	79.5
*t*_*start*_	72.1
*t*_*end*_	4.9
*α*	0.25
*β*	0.77
*t*_*illum*_ (2)	22.5
*t*_*illum*_ (3)	0.57

Using our optimization method, we could not increase the classification accuracy above 82% for any considered geometry within 1,000 optimization steps. However, the accuracy can be increased if one combines answers of different classifiers using the voting strategy. We considered three classifiers that showed the highest accuracy. They were based on 3, 5, and 6 oscillators. The same record was processed by all classifiers, and the majority verdict was taken as the answer. Such a method gave only one mistakenly diagnosed case for 45 schizophrenic records from *T*_*S*_. The classification accuracy for healthy patients (39 records in *T*_*S*_) was lower, and 10 such cases were misdiagnosed. Therefore, the overall accuracy of classification increased to 86.9%.

## 5 Conclusion and Discussion

One of the most significant challenges of civilization is how to use Artificial Intelligence (AI) for various life-inspired problems AI (2022). AI techniques can be beneficial for medical applications where the knowledge is accumulated as information on previously cured cases. To diagnose a new patient, one should search for similarities with the previous ones. Here we presented an application of AI methods for designing a system that can help to diagnose schizophrenia. We assumed that schizophrenia could be detected by a chemical oscillator network that analyses EEG signals recorded from electrodes located on a patient scalp.

We considered a few information processing networks characterized by different numbers of nodes (c.f. Figure 2). We think that networks of interacting chemical oscillators represent more realistic models of biological neural computing than typical artificial neural networks with arbitrarily selected activity rules Kay (2003). Their time evolution is described by realistic kinetic equations that model specific nonlinear chemical reactions. Here we used the Oregonator model for the Belousov-Zhabotinsky reaction. We believe that other, more realistic models Vanag and Epstein (2009) of chemical evolution lead to qualitatively similar results. It seems that oscillator networks require a smaller number of nodes than standard neural networks to achieve the same accuracy. The results presented in Gorecki and Bose (2020) indicate that just three oscillators can solve a geometrical problem of how a point from a unit square is located with respect to a disk placed at the square center with 95% accuracy. The fact that just a few oscillators can perform a complex information processing function, confirmed by the results for schizophrenia diagnostics reported in this paper, opens the door for experimental realization of chemical instant machines with systems of interacting oscillators reported in the literature Juan Manuel et al. (2020); Vanag and Yasuk (2018); Vanag. (2019a); Proskurkin et al. (2019); Gizynski and Gorecki (2017b).

We expected that the schizophrenia diagnosis accuracy increases with the network size. However, the networks formed of 3, 5, and 6 oscillators gave 82% of correct answers for cases included in the training dataset. On the other hand, this number is much higher than given by standard classification methods included as options in the Clasiffy procedure of the Mathematica program Mathematica (2021). If we apply this procedure to the training dataset *T*_*S*_, the highest classification accuracy (76.2%) is obtained for the GradientBoostedTrees method, whereas the NeuralNetwork option leads to 73.8% accuracy. The accuracy of schizophrenia diagnosis using chemical oscillator networks increased to 86.9% if three networks process a case and the majority rule is used to select the final answer. The fact that larger networks did not produce better results than the small ones could be related to inefficient optimization for a large number of parameters that were taken into account. The problem can be overcome by a larger population of classifiers and a larger number of optimization steps. However, both methods increase the numerical complexity of the optimization. It is also worth mentioning that the recent study on the application of machine learning methods for schizophrenia detection from textual input Wawer et al. (2022) reported ∼ 80% accuracy on a sample of 94 people (47 ill and 47 healthy).

Although the presented results are encouraging, datasets with a larger number of patient data are important for further studies. A dataset with a large number of patient records can be separated into a training dataset of a few thousand cases and much larger testing ones Gorecki and Bose (2020) that is independent of the training one. The separation of records between the training and the testing datasets can be done in many different ways. By selecting different training datasets, one can verify the stability of the schizophrenia diagnostic classifier with respect to different training. The observation that classifiers with similar parameters are obtained for different training datasets confirms that the diagnostics are unbiased by selecting a training dataset.

The presented classification method is based on many assumptions. All of them can be lifted in search of the best network for the schizophrenia diagnosis.

The optimization of interactions in the medium can be directly included in the optimization program. The presented results were obtained assuming that interactions between oscillators were fixed, as shown in Figure 2. The information about interactions was included in equations describing the time evolution of oscillators as the binary parameters *s*_*i*,*j*_ in Eq. 14. The values of these parameters can be included in classifier optimization. The network model includes the activatory coupling between oscillators. It means that an excited oscillator can speed up the excitations of the other oscillators that interact with it. Such coupling is observed, for example, in droplets containing reagents of BZ reaction. Alternatively, one can consider a medium with inhibitory coupling where excitation of one oscillator slows down the activity of those oscillators that interact with it Vanag and Yasuk (2018); Vanag. (2019b); Proskurkin et al. (2019). Allowing for different types of coupling within a single network can help to identify the best medium for a given computing task.

We assumed that the output could be related to the number of activator maxima observed at a specific oscillator. However, one can consider alternative methods of extracting information from network evolution Borst and Theunissen (1999); Zhang et al. (2014). For example, the output can be related to a pair of numbers of activator excitations recorded on two selected oscillators Gorecki (2020).

It is anticipated that the accuracy of diagnosis should improve if the information on signals recorded on more than two electrodes is included in the input. The presented optimization algorithm can be easily modified to do this if one includes input oscillators of any important signal into the network. If additional signals do not increase the *Fitness*, then networks with the inputs of irrelevant signals will vanish from the population. Moreover, to improve the accuracy of a large oscillator network, one should consider different decay rates *α* at different oscillators and different activator transfer rates *β* for individual couplings. Of course, it results in a significant increase in the number of parameters undergoing optimization.

Future studies should reveal if the generalizations of classifiers as described above can significantly increase their accuracy of schizophrenia diagnosis if compared with the classifiers presented in this paper. After a successful classifier optimization is completed, its application does not require significant computing power. There are just two steps of the algorithm: 1) normalization of patient data with parameters (*μ*, *σ*) obtained for the training dataset and 2) numerical solution of differential Eqs 14 and 15 and activator maxima counting. A modern laptop needs a few seconds to execute these tasks. The whole procedure can be incorporated into EEG equipment software or distributed as a laptop or smartphone application.

## Data Availability

The original contributions presented in the study are included in the article/Supplementary Material, further inquiries can be directed to the corresponding author.
